# Enhancing AI Readiness in Pediatric Surgery: Impact of a Targeted Workshop on Knowledge and Competencies

**DOI:** 10.1055/a-2650-6603

**Published:** 2025-07-24

**Authors:** Holger Till, Hesham Elsayed, Georg Singer, Beate Obermüller, Tristan Till, Richard Gnatzy, Sebastian Tschauner

**Affiliations:** 1 Department of Pediatric and Adolescent Surgery, Medical University of Graz, Graz, Austria; 2 College of Computing and Data Science (CCDS), Nanyang Technological University, Singapore, Singapore; 3 Department of Pediatric Surgery, Leipzig University, Leipzig, Germany; 4 Division of Pediatric Radiology, Department of Radiology, Medical University of Graz, Graz, Austria

**Keywords:** artificial intelligence, machine learning, competencies, educational needs, pediatric surgery

## Abstract

**Introduction:**

Despite an awareness of the transformative potential of artificial intelligence (AI) in health care, its development in pediatric surgery seems slow. One major reason may be a lack of formal AI training. This study assesses the basic AI knowledge and the effectiveness of AI workshops (AI-WS).

**Materials and Methods:**

Four AI-WS were held at the International Academy of Pediatric Surgery 2024. Topics included AI principles, real-time algorithm training, and potential AI applications in pediatric surgery. Self-developed surveys consisting of eight pre-WS and nine post-WS questions were conducted, focusing on participants' AI competencies, usage, educational needs, barriers, and future perspectives.

**Results:**

Out of 57 pediatric surgeons, 53 completed both surveys. None had formal AI training. Although 90% were familiar with AI in diagnostic imaging, most had only basic knowledge of AI technology. After the workshop, participants reported a significant increase in the general understanding of AI/machine learning (ML) ( *p* < 0.001). 96% stated that they were better informed about AI/ML applications for clinical practice; 83% expressed interest in further AI training; 91% believed that AI will be more integrated into clinical practice; and over 80% anticipated that AI will improve patient outcomes.

**Conclusion:**

The AI-WS effectively enhanced pediatric surgeons' AI knowledge and their readiness to adopt AI technologies. Even though our study is limited by the relatively low sample size and a potential selection bias, our results still highlight the importance of targeted education in preparing health care professionals for AI integration. The long-term sustainability of knowledge gains, however, has to be examined in further studies.

## Introduction

Artificial intelligence (AI) has emerged as a transformative force across multiple medical specialties, offering unprecedented opportunities to improve diagnostic accuracy, enhance clinical decision-making, and ultimately elevate patient outcomes. 1 2 However, the successful integration of AI into clinical practice is contingent on the preparedness and competencies of health care professionals. 2 3 Despite its growing potential, many clinicians, including pediatric surgeons, have not received formal training in AI, leaving them without the foundational knowledge necessary to engage meaningfully with these emerging technologies. 4 5 Nevertheless, AI and machine learning (ML) applications are of specific interest to pediatric surgeons due to the limited case numbers of treated diseases, lack of pediatric-specific algorithms, ethical concerns, and data safety.

The knowledge gap of clinicians could be narrowed by formal curriculum changes, online modular courses, mentorship or fellowship programs, but also workshops held at educational or scientific conferences. 6 7 The demand for a structured AI training for undergraduate students, but also pediatric endosurgeons, has already been shown in previous publications. 4 8 Academic societies and professional conferences are uniquely positioned to assess the current level of AI knowledge and utilization among their members. These institutions, with their extensive networks and access to health care professionals, can serve as critical platforms for addressing AI competency gaps through targeted educational initiatives such as workshops at conferences. By offering specialized AI training, academic societies can play a key role in closing the knowledge gaps, fostering greater acceptance of AI, and facilitating its effective implementation within clinical practice. 4

To explore the feasibility and impact of such educational interventions, we conducted a pre- and postworkshop survey aimed at pediatric surgeons from Germany, Switzerland, and Austria. This study evaluates the efficacy of AI competency workshops (AI-WS) in enhancing AI knowledge and attitudes among pediatric surgeons, with a focus on bridging the knowledge gap and promoting the integration of AI tools into pediatric surgery. We hypothesized that a formal workshop may increase pediatric surgeons' knowledge regarding AI and their readiness to adopt AI technologies.

## Materials and Methods

Following approval by the Ethics Committee of the Medical University of Graz (protocol code 1292/2024), four 90-minute-long AI-WS sessions were conducted during the 2024 International Academy of Pediatric Surgery (AKIC) with up to 15 participants in each of the sessions (57 participants in total). AKIC is the most important yearly postgraduate training program in pediatric surgery and is meant to prepare residents specifically for the board exam in pediatric surgery. Therefore, the participants were senior residents preparing for their final exams in pediatric surgery.

All four sessions were given by a pediatric surgeon with 4 years and a pediatric radiologist with 9 years of experience in AI/ML and covered fundamental AI/ML principles such as image classification, object detection, image segmentation, dataset preparation, data cleaning, data preprocessing and augmentation, real-time training with an ML algorithm, and an outlook on future AI support systems in laparoscopic surgery.

Surveys were administered online to all participants before and after each session using LimeSurvey and consisted of eight preworkshop and nine postworkshop questions ( Supplementary Table S1 [available in the online version only]). The surveys consisted of single-choice and multiple-choice questions designed to assess participants' baseline knowledge and postsession changes in understanding and attitudes toward AI/ML in clinical settings. The items were specifically developed for this study based on a previously published study, 4 as no standardized instruments currently exist for this emerging field in pediatric surgery. While the surveys were not formally validated or tested for statistical reliability, they were piloted several times within our Department to ensure clarity and relevance. Participation was voluntary and anonymous.

### Statistical Analysis

Quantitative data were analyzed using descriptive statistics, including response rates and frequency distributions. Categorical data comparisons were performed with the chi-square test. A *p* -value <0.05 was considered statistically significant.

## Results

**Fig. 1:**
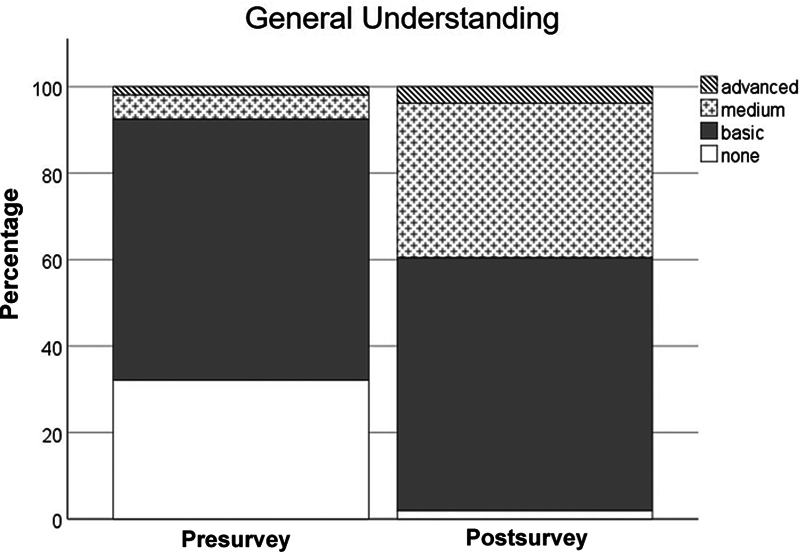
Comparison of the pre- and postsurvey answers to the question “How would you rate your general understanding of AI/ML?” *p* < 0.001 (chi-square test). AI, artificial intelligence; ML, machine learning.

In total, 53 out of 57 participants provided complete answers both in the pre- and postworkshop surveys. The comparison of the pre- and postworkshop survey revealed a statistically significant increase in the general understanding of AI/ML ( *χ* ^2^ [Df 3, *n* = 106] = 26.2, *p* < 0.001; Fig. 1 ).

### Preworkshop Survey

None of the 53 respondents has received a formal training in AI/ML. While *n* = 23 (43%) of the participants stated that they do not use ChatGPT or other AI tools, *n* = 3 (6%) use these tools for work, *n* = 12 (23%) in private life, and *n* = 15 (28%) for both.

**Fig. 2:**
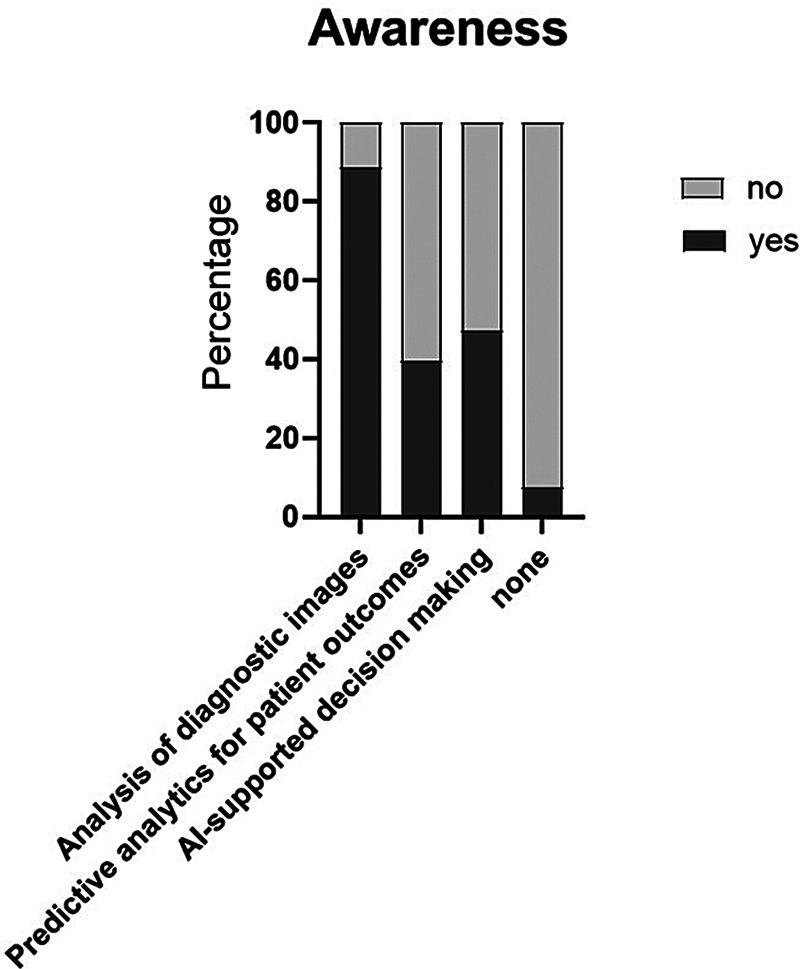
Applications of ML in health care or clinical settings the participants are aware of. AI, artificial intelligence; ML, machine learning.

Figure 2 shows the applications of ML in health care or clinical settings that the participants are aware of. Almost 90% are aware of ML used for the analysis of diagnostic images.

More than three-quarters ( *n* = 43, 81%) of the 53 respondents answered that they do not use ML in their practice and research; *n* = 7 (13%) use ML, and *n* = 3 (6%) were unsure. The vast majority ( *n* = 45, 85%) stated that they believe ML can improve patient care or clinical outcomes; *n* = 8 (15%) were unsure. About *n* = 49 (93%) of the participants would be interested in receiving further training on the applications of AI/ML in their field. The most important challenges in implementing ML in clinical practice considered by the respondents were ethical issues ( *n* = 22, 41%), lack of understanding ( *n* = 20, 38%), lack of resources ( *n* = 9, 17%), and others ( *n* = 2, 4%).

### Postworkshop Survey

51 out of 53 participants (96%) stated that—after the workshop—they feel better informed about specific applications of ML for everyday clinical practice; 2 answered this question with no (4%). More than two-thirds ( *n* = 36, 68%) are more inclined to use ML tools in everyday clinical practice or in research following the workshops, *n* = 14 (26%) were unsure, and *n* = 3 (6%) answered this question with no. The workshops influenced the attitude toward AI/ML in health care positively in *n* = 39 (74%) and neutral in *n* = 14 (26%) respondents.

The vast majority ( *n* = 48, 91%) think that AI/ML will be more integrated into everyday clinical practice in the next 5 years, *n* = 4 (7%) were unsure, and *n* = 1 (2%) answered this question with no. *n* = 44 participants (83%) were interested in further training on AI/ML after the workshops.

**Fig. 3:**
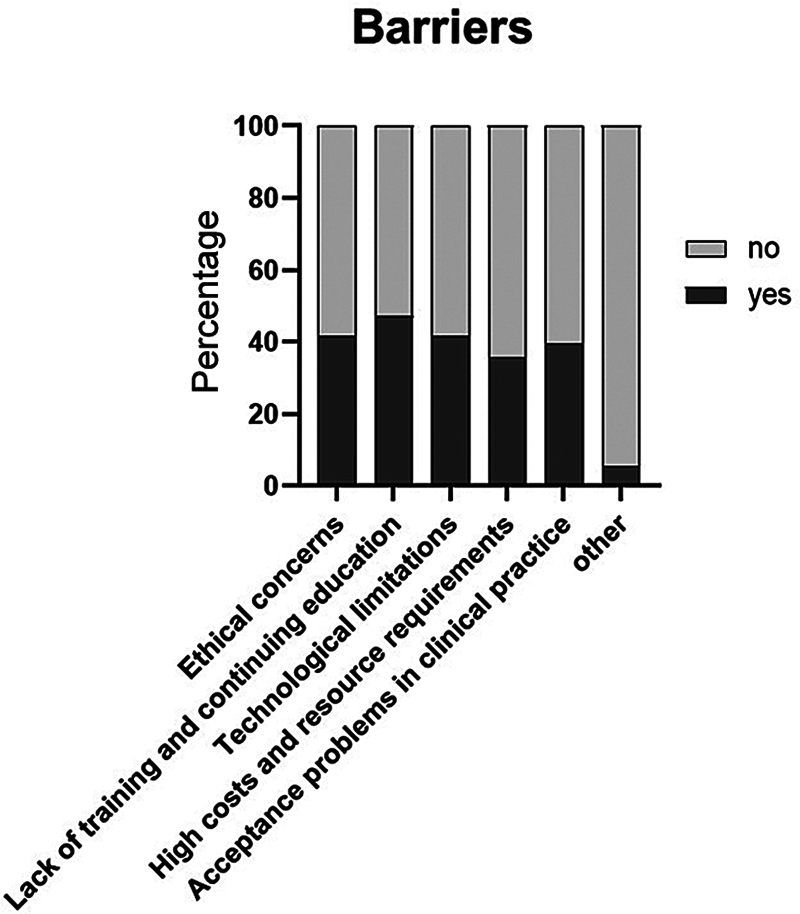
Assessment of the key barriers in using and implementing ML in health care. ML, machine learning.

Figure 3 shows the participants' assessment of the key barriers to using and implementing ML in health care.

The impact the participants expect AI/ML to have in their field in the future consisted of special applications ( *n* = 26, 49%), transformation of daily routines ( *n* = 20, 38%), unsure ( *n* = 5, 9%), and none ( *n* = 2, 4%).

*n* = 44 (83%) respondents think that ML will lead to better patient outcomes in their practice, *n* = 2 (4%) do not think so, and *n* = 7 (13%) were unsure.

## Discussion

This study provides certain evidence that structured AI/ML workshops (AI-WS) can enhance the knowledge and attitudes of pediatric surgeons toward AI in clinical practice. The results from the pre- and postworkshop surveys highlight the effectiveness of targeted AI education in bridging the knowledge gap, fostering greater acceptance of AI technologies, and increasing enthusiasm for further AI training. These findings support the role that academic societies and professional workshops could play in preparing surgeons for the integration of AI into their practice. 4 9 10

### Knowledge Improvement

Before the AI-WS, none of the participants had received formal AI/ML training, yet nearly 90% of them were aware of ML's application in diagnostic imaging. This demonstrates a general awareness of AI but without a deep, actionable understanding. The workshop's success is particularly evident in the significant improvement in participants' self-assessed general understanding of AI posttraining. The structured approach to AI education, including both theoretical and practical components, may have contributed to this shift. After the workshop, the participants reported a deeper understanding of how AI/ML could be applied in their field, not just in imaging, but also in broader surgical contexts, such as decision support and patient outcome prediction. Nevertheless, whether this understanding and enthusiasm are lasting is still unclear and subject to further studies.

### Attitudinal Change and Future Integration of Artificial Intelligence/Machine Learning

One of the most notable outcomes of this study is the shift in attitudes toward AI/ML integration in clinical practice. Before the workshop, while participants recognized the potential of AI in health care, many expressed concerns due to a lack of understanding of AI/ML and how it could be practically applied to improve or streamline everyday clinical tasks. This lack of clarity contributed to their initial hesitations, as well as the ethical implications of using AI/ML. Postworkshop, 91% of participants expressed the belief that AI/ML would be increasingly integrated into clinical practice in the next 5 years, reflecting an optimistic outlook on the future of AI in pediatric surgery. 11 12

This change in perspective is crucial, as attitudes often determine the pace and success of AI adoption in clinical settings. By providing participants with not only the foundational knowledge but also the practical tools to engage with AI technologies, the AI-WS may have promoted a more optimistic mindset toward AI integration into clinical practice. This shift suggests that well-designed workshops may effectively counteract initial skepticism, helping participants see AI as a valuable tool to enhance their practice, rather than a threat to their professional autonomy or skills. 13 However, it is not known whether the abovementioned shift is due to a response bias of the participants wanting “to please” the organizers of the workshops.

### Enthusiasm for Continued Learning

Participants expressed an interest in further AI/ML training, with 83% of participants indicating a desire to pursue more advanced education in the field. This high demand for continued education in AI/ML highlights the participants' recognition of AI's importance and their desire to stay at the forefront of technological advances in surgery. The willingness of pediatric surgeons to engage with AI beyond a single workshop suggests that once the foundational barriers of knowledge and understanding are addressed, pediatric surgeons are eager to explore more advanced applications and tools. This further emphasizes the need for continued, accessible educational opportunities in AI for clinicians. 10 Nevertheless, it remains unclear whether the participants truly will engage in continued learning regarding AI/ML or if the enthusiasm is only a short-term “conference effect.”

### Perceived Impact on Patient Outcomes

Another outcome from the workshop was the participants' confidence that AI/ML would improve patient outcomes. Over 80% of respondents indicated that they believed AI/ML technologies could have a beneficial impact on their practice, particularly in improving precision, decision-making, and patient care. This belief in AI's potential to enhance clinical outcomes is crucial for successful integration. When surgeons perceive AI as a tool that can directly contribute to better patient care, they are more likely to adopt and utilize it effectively in their practice. Moreover, this aligns with findings in other medical specialties, where AI has been shown to improve diagnostic accuracy, surgical precision, and postoperative care outcomes. 11 14 15 16 17

### Ethical and Operational Concerns

Despite the positive shift in knowledge and attitudes, ethical concerns about AI remain a significant challenge, as noted in the preworkshop survey. Participants expressed particular anxiety about potential data biases and the erosion of surgeon autonomy. These concerns are valid and reflect broader societal debates about the ethical implications of AI in medicine. 18 19

The AI-WS addressed some of these concerns by providing an overview of AI systems' design and implementation, including discussions on data quality, transparency, and regulation. However, further education on the ethical considerations surrounding AI is still crucial. 11 Surgeons need to feel confident that AI systems are not just technically reliable but also ethically sound. Incorporating detailed discussions on the ethical and legal frameworks governing AI in future workshops, webinars, or other modalities will be essential in addressing these concerns and fostering broader acceptance. Additionally, emphasizing that AI is designed to augment, not replace, human decision-making can help alleviate fears of diminished professional autonomy. 20

### The Role of Academic Societies in Artificial Intelligence Education

Academic societies can play a leading role in the integration of AI education into the medical profession. These societies are uniquely positioned to develop and implement educational curricula that focus on both the theoretical understanding and practical application of AI/ML in clinical settings. As demonstrated by our results, such initiatives can bridge critical knowledge gaps and significantly influence attitudes toward AI adoption.

By offering structured, evidence-based AI training programs, academic societies could ensure that surgeons are not only familiar with emerging AI technologies but are also empowered to actively contribute to their development. Academic societies can leverage their established networks to bring together multidisciplinary teams, enabling surgeons, researchers, and AI experts to collaborate on the creation of AI tools that are specifically tailored to the unique needs of pediatric surgery. 11

Furthermore, incorporating AI education into ongoing professional development programs will ensure that surgeons are better prepared to integrate new AI tools into their practice as they evolve.

## Limitations and Future Directions

While this study demonstrates the efficacy of the AI-WS, it certainly has several limitations. First, the survey sample consisted of only 53 pediatric surgery residents, all of whom voluntarily participated in a professional conference, which may have skewed the results in terms of a selection bias toward individuals already interested in or aware of AI. Moreover, the absence of a control group also makes it unclear whether the improvement is due to the intervention or other external factors such as general enthusiasm. Future research should therefore include a broader, more diverse sample, including also more experienced clinicians from community hospitals and private practices as well as a control group, to assess the generalizability of these findings across various practice settings.

Additionally, the study did not evaluate the long-term retention of AI knowledge or the actual implementation of AI tools in clinical practice. Future studies should follow up with participants after a longer period to assess how well the knowledge gained from the workshop translates into real-world applications. It would also be valuable to examine the impact of AI/ML training on clinical outcomes, as this would provide a direct measure of how such workshops influence patient care.

Moreover, the nature of the anonymized pre- and postworkshop questions did not allow us to calculate more *p* -values, and the majority of our results are therefore reported as descriptive statistics. Asking the same set of questions before, directly after, and after a longer time period could assess the lasting effect of AI-WS in future studies.

## Conclusion

This study provides certain evidence that AI/ML education through targeted workshops may enhance pediatric surgeons' understanding of AI technologies, shift their attitudes toward AI integration and increase their enthusiasm for further learning. The results demonstrate that such workshops can play a crucial role in preparing surgeons to adopt AI tools. Nevertheless, it has to be taken into account that we have used self-assessment data and an unvalidated questionnaire. Moreover, the role of other modalities, such as webinars or review articles, is subject to future studies.

Moving forward, academic societies should continue to prioritize AI education, ensuring that surgeons are equipped with the knowledge, skills, and confidence to engage with AI in their practice. As AI continues to evolve, so too should the educational opportunities provided to health care professionals, ensuring that they remain at the cutting edge of medical innovation.
