# Supplementary material for: Enhancing AI Readiness in Pediatric Surgery: Impact of a Targeted Workshop on Knowledge and Competencies
Source: Eur J Pediatr Surg. 2025 Jul 24;36(2):125–9. doi: 10.1055/a-2650-6603 (PMC12965792; doi:10.1055/a-2650-6603)
Supplement: Supplementary file 1 — Supplementary Material [file 10-1055-a-2650-6603-s2025027195oa.pdf]

Pre-Workshop Survey

1. How would you assess your general understanding of AI? (Single Choice)	• None
	• Basic
	• Medium
	• Advanced

2. Have you received formal training in Artificial Intelligence (AI)? (Single Choice)	• Yes
	• No

3. Do you use ChatGPT or other AI tools in your personal or professional life? (Single Choice)	• Yes, but only in my professional life
	• Yes, but only in my private life
	• Yes, both in my professional and personal life
	• No

4. Which of the following applications of ML in healthcare or clinical settings are you familiar with? (Multiple Choice)	• Analysis of diagnostic images
	• Predictive analytics for patient outcomes
	• AI-supported decision-making
	• None of the above

5. Are you currently using AI in your practice or research? (Single Choice)	• Yes
	• No
	• Not sure

6. Do you believe that AI/ML can improve patient care or clinical outcomes? (Single Choice)	• Yes
	• No
	• Not sure

7. Would you be interested in further training on the applications of AI/ML in your field? (Single Choice)	• Yes
	• No

8. What do you think is the biggest challenge in implementing AI/ML in clinical practice? (Single Choice)	• Lack of resources
	• Lack of understanding of AI/ML
	• Ethical issues (e.g., bias, data privacy)
	• Other (please specify)

Post-Workshop Survey

1. How would you now assess your understanding of AI after the workshop?	• None
	• Basic
	• Medium
	• Advanced

2. Do you feel better informed about specific AI applications for clinical practice after the workshop? (Single Choice)	• Yes
	• No
	• Not sure

3. Are you now more inclined to use AI/ML tools in clinical practice or research after attending the workshop? (Single Choice)	• Yes
	• No
	• Not sure

4. How has the workshop influenced your attitude towards AI in healthcare? (Single Choice)	• More positive
	• Neutral
	• Negative

5. Do you believe AI will be more strongly integrated into clinical practice within the next five years? (Single Choice)	• Yes
	• No
	• Not sure

6. Would you like to participate in further AI training after the workshop? (Single Choice)	• Yes
	• No

7. What do you see as the main barriers to the adoption and implementation of AI in healthcare? (Multiple Choice)	• Ethical concerns (e.g., data privacy)
	• Lack of training and continuing education
	• Technological limitations
	• High costs and resource requirements
	• Acceptance problems in clinical practice
	• Other (please specify)

8. What impact do you expect AI to have in your field in the future? (Single Choice)	• Transformation of daily routines
	• Limited to special applications
	• no impact
	• Unsure

9. Do you believe AI/ML will lead to better patient outcomes in your practice? (Single Choice)	• Yes
	• No
	• Not Sure
